# Development of an Internet-based system to guide and telemonitor hearing aid users

**DOI:** 10.1590/2317-1782/20232022162en

**Published:** 2023-12-04

**Authors:** Andrea Soares, Katia de Almeida

**Affiliations:** 1 Faculdade de Ciências Médicas da Santa Casa de São Paulo – FCMSCSP - São Paulo (SP), Brasil.

**Keywords:** Hearing Aids, Elderly, Adults, Distance Counseling, Internet-Based Intervention, Telehealth, Technological Development,Patient Satisfaction

## Abstract

**Purpose:**

To develop and verify the usability of an internet-based system for telemonitoring and guidance of the hearing aid user as well as monitoring the long-term performance in a pilot group.

**Methods:**

The system “I can hear, but I can’t understand” was developed based on recommendations in the literature regarding layout, design, and content for guidance and advice. Three stages were followed: planning, design and content development, and pilot testing. The sample consisted of 43 adults and older adults with any type and degree of hearing loss, who had been regularly using a hearing aid for at least 30 days and at most 24 months, with reading skills and no evidence of cognitive impairments. The individuals were followed up for 8 to 12 months. The users’ performance was monitored with the Speech, Spatial and Qualities of Hearing Scale. The usability of this material was assessed with the System Usability Scale.

**Results:**

Improved performance and increased self-reported daily use of the hearing aid were observed after the period of guidance and telemonitoring via the system for all research participants. In all analyzes of the SUS scale, it was possible to observe a performance superior to 70 points, demonstrating good usability of the system. In the analysis of the performance of the SSQ, in the three moments of the research, a positive response was observed in all domains, thus showing progress in the use of hearing aids, with significant data for the domain of Hearing Speech.

**Conclusion:**

The system “I can hear, but I can’t understand” proved to be an easy-to-use and effective tool to telemonitor hearing aid users.

## INTRODUCTION

Fitting hearing aids is a multiple complex process that is not limited only to the period of tests and the choice of the device. Many pieces of research demonstrate that the absence of guidance and inadequate follow-up lead people to abandon the use of hearing aids^(1)^. It has been estimated that the non-use of already acquired hearing aids in adults reaches up to 24% of users^(2,3)^.

Various new or experienced users report difficulties using the devices, dissatisfaction using them, and effort to retain the guidance they received. Some subjects do not look for help because they do not realize they are having problems or may have better results with the use of amplification^(4)^. The most common complaints among users of hearing aids are related to speaking on the telephone, understanding speech in noise, correctly putting on batteries, cleaning, identifying the side of the device, caring for the device in terms of humidity, and turning it on^(4,5)^. These handling problems often have the greatest impact on successful fitting – they are also the most difficult ones to solve^(6)^.

Speech-language-hearing therapists must provide adequate training, counseling, and periodical monitoring to all individuals to improve their experience and increase effectiveness in the hearing aid fitting process. It must be highlighted that, besides verbal explanations, professionals must provide material with appropriate language, considering the person's capacity to read^(7)^ and educational software usability principles^(8)^.

Following up on the user’s performance is essential to ensure good hearing aid fitting. Self-assessment questionnaires evaluate self-perceived hearing difficulties, functioning limitations, participation restrictions, and the users’ benefits and satisfaction. Using these tools in healthcare is a differential that may provide much qualitative information to help the hearing aid fitting process. The currently most used such questionnaires include the Speech, Spatial and Qualities of Hearing Scale – SSQ. SSQ is a self-reported hearing-incapacity measure that aims to assess patients’ participation in hearing activities and subjective experiences and quantify hearing inabilities in communication situations such as directional hearing, ease of hearing, and clarity of sounds.

Traditional models that provide hearing rehabilitation services focus on in-person tests with device adjustments and counseling. However, they require many visits to the place of care. Thus, patients with difficulties attending such consultations in person do not have adequate follow-up. Telehealth is an alternative to increase patients’ access to guidance. Such a care model is offered to patients that are near the health professional but choose to have remote care as an option due to convenience. Blended healthcare, adding telehealth care to in-person care, has also proved to be effective and satisfy patients, both to validate tools and rehabilitate hearing, focused on patient satisfaction and experience^(9)^, with similar results between new and experienced users, regardless of the mode of care^(10)^.

Telecare reduces the cost of guidance, enabling better access to care and better satisfaction results among hearing aid users. It increases the appropriation and effectiveness of guidance and clinical practice (especially in terms of technological development and technical and clinical validation), optimizes strategies to provide care^(11)^, and furnishes patient-centered telehealth^(2,12-15)^.

Usability assesses the quality of the users’ experience interacting with technology^(16)^, verifying whether the project can be used by users to reach specific objectives effectively, efficiently, and satisfactorily^(17)^. Nielsen^(18)^ defined five components of usability quality: learning, efficiency, memorization capacity, tolerance to errors, and satisfaction. To Brooke^(19)^ usability parameters like effectiveness, efficiency, and learning capacity must be objectively measured or quantified, whereas subjective measures (user satisfaction) are more generically assessed with general attitude questionnaires or scales such as the System Usability Scale (SUS).

Usability tests provide valuable insight into the experiences and process of using the material. They can also be used to explain the results of measures such as self-reported benefits and satisfaction^(13,19)^. Much discussion on innovations in hearing health concentrate on patients’ unmet needs and the resources to meet them, but no discussion on hearing technology will be complete until the competencies of the person using the technology are considered^(13)^.

Given the above, this study aimed to develop and verify the usability of an Internet-based system to telemonitor and guide hearing aid users and monitor long-term performance in a pilot group.

## METHODS

This study had three stages: planning, developing the design and contents, and pilot testing. The decision to develop an online system rather than an application was based on both the high cost of the application and the difficulty maintaining the guidance material and mobile operating systems updated. The system can be accessed from computers and smartphones, thus enabling access to more people.

The material aims to be a reusable tool, with brief visual and interactive information. The whole material was developed based on usability and design principles, focused on topics related to guidance, counseling, and follow-up of hearing-impaired adults and older adults who use hearing aids.

The system is called “I can hear, but I can’t understand”, available from site “Escuto mas não entendo” and registered in INPI (Portuguese for National Institute of Industrial Property) under process number 512022001680-0. The initial page has six guidance texts, and the restricted area has another 26 texts distributed into the following divisions: 1. Institutional Area; 2. Room to insert informative texts, named News; 3. Restricted area in which patients are registered by their tutor for healthcare; 4. The patient’s room, where they can exchange messages asynchronously with professionals; 5. Self-assessment area, in which patients respond to the SSQ-12 questionnaire; and 6. Guidance area. The guidance content in the system can only be accessed with the patient’s login and password, complying with the guidelines in the General Personal Data Protection Law (LGPD).

The guidance area was divided into four sections: hearing and hearing loss, handling and caring for hearing aids, communication strategies, and frequently asked questions. Also. 29 images (drawings, illustrations, and pictures) and eight short videos lasting less than 1 minute each were developed to illustrate the 26 guidance texts to make understanding easier for users, aiming to increase guidance material readability. The channel “Escuto mas não entendo” was created on YouTube for videos to be available to the public. The term hearing aids was used in all guidance materials aimed at hearing aid users for being more popular and known by the lay public.

This project was analyzed and approved by the Research Ethics Committee of the *Santa Casa de São Paulo*, under number 3.443.374. Individuals who voluntarily agreed to participate in the study signed an informed consent form. Data were collected at a private clinic in the city of São Paulo. The sample comprised 43 individuals aged 27 to 87 years of both sexes, with any type and degree of bilateral or unilateral hearing loss, classified according to the reference by WHO^(20)^. The inclusion criteria were as follows: literate hearing aids users, who had been using it for up to 24 months; new users, who had been using it for at least 30 days; without important visual problems; with good reading skills; and no evidence of cognitive impairments. The exclusion criteria were the lack of access to the Internet and no regular use of hearing aids.

In the in-person visit, the patient’s medical history was surveyed to find information on educational attainment, professional activity, regular hearing aid use, and associated difficulties.

The study considered active individuals who had any paid occupation, while the other subjects were considered inactive. In the same visit, the researcher also assessed the daily time of views recorded in the software and reported by the patient. All patients were submitted to the same fitting protocol to ensure adequate hearing aid programming at the beginning of the collection.

The users’ performance was monitored with the 12-question SSQ questionnaire, short version, translated into Brazilian Portuguese^(21,22)^. Experienced users answered the questionnaire based on their performance with the hearing aids at the moment – which was called SSQ-1. New users answered SSQ 1 based on the performance with the hearing aids 30 days after fitting. Those who received intervention answered the SSQ questionnaire after teleconsultations – which was called SSQ-2. At the end of the telemonitoring period, participants were asked to answer SSQ again in the system to assess the benefit and satisfaction after then. This questionnaire application was called SSQ-3. A scale was used with scores ranging from 0 to 10, in which 10 indicated perfect performance, and 0 indicated great difficulty at the time of the answer.

All participants were followed up for a minimum of 8 and a maximum of 12 months to assess the effectiveness of the material with a participative approach throughout telemonitoring. All study participants were instructed to use the asynchronous message session whenever they needed to report difficulties or answer questions. The professional answered the messages asynchronously, sending specific guidance material from the system; when necessary, they scheduled a video call teleconsultation appointment.

By the end of this period, the time of daily hearing aid use was recorded as reported by the patient, who attended an in-person appointment when necessary. The final time of use, measured with data logging, could not be measured due to the period of social isolation and restrictions during the pandemic.

To assess the research system usability, each participant was sent SUS^(19)^ along with a questionnaire to report what area in the system they accessed the most. Both instruments were sent at the end of the telemonitoring period Via Google Forms. This analysis aimed to assess system exploration (doing tasks and ease of access), use effectivity (capacity to find information inside the system), understanding instructions for use described in the system, use efficiency (questions on the levels of difficulty and comfort while using the information in the system), and user satisfaction (questions on subjective reactions of users after using the system).

SUS was analyzed according to the approach by Lewis and Sauro^(23)^ – i.e., analyzing the whole questionnaire, rather than the individual questions. Each item score ranged from 0 to 4. In items with positive words (odd numbers), the score contribution is its position on the scale minus 1, while in items with negative words (even numbers), the score contribution is 5 minus its position on the scale. The overall score is obtained by multiplying the sum of score contributions by 2.5, producing an index that ranges from 0 (usability perceived as very poor) to 100 (usability perceived as excellent); 68 is considered average usability, and 80, above average^(23,24)^.

Google Analytics data were used to verify the most accessed pages in the system and the most used devices to access them throughout telemonitoring. To find whether answers were similar to the users’ perception, users were sent a questionnaire developed for this research with four items corresponding to the areas of the system (hearing and hearing loss, handling and caring for the hearing aid, communication strategies, and frequently asked questions). Users were asked to indicate which area they accessed the most during telemonitoring – only one area could be checked. Participants were allocated into groups for this analysis according to their age (younger adults = up to 60 years old; older adult = 60 years or older), occupation (active = 23 users; inactive = 20 users), and time of hearing aid use (new users = 17; experienced users = 26). A descriptive analysis was conducted for qualitative variables: sex, educational attainment, degree and type of hearing loss, occupation, and hearing aid characteristics. statistical tests were performed in SPSS 25.0.

## RESULTS

The sample comprised 43 individuals, distributed into two groups according to their experience with hearing aids: 26 experienced users (14 women with a mean age of 53.8 years and 12 men with a mean age of 46.2 years), having used them for up to 2 years; and 17 new users (8 women with a mean age of 47.1 years and 9 with a mean age of 52.9 years). No statistically significant differences were found between the groups regarding sex and age (p = 0.663, chi-square test). The subject's educational attainment ranged from high school graduates to postgraduates with no significant differences between the groups (p = 0.461).

The sample’s hearing thresholds were similar between the groups regarding hearing loss laterality ((p = 1, Fisher’s exact test), hearing aid use (p = 0.376, Fisher’s exact test) (unilateral x bilateral), and type of hearing loss in the right (p = 0.227, chi-square test) and left ear (p = 0.348, chi-square test). Thus, the sample had predominantly bilateral sensorineural hearing loss in both years with bilaterally fitted hearing aids.

There were statistically significant differences between the groups regarding occupation (p = 0.002) in the chi-square test analysis between active and inactive participants. The group of experienced users mostly had professionally inactive individuals 65.4% (n = 17), whereas the group of new users mostly had professionally active individuals 82.4% (n = 14).

The users accessed the system 91% of the time, regardless of their age, while the other 9% refer to access by relatives or caregivers. Most accesses were from computers (69.95%) and mobiles (29.58%), whereas only a small portion was from tablets (0.46%) throughout telemonitoring.

**Figure 1:**
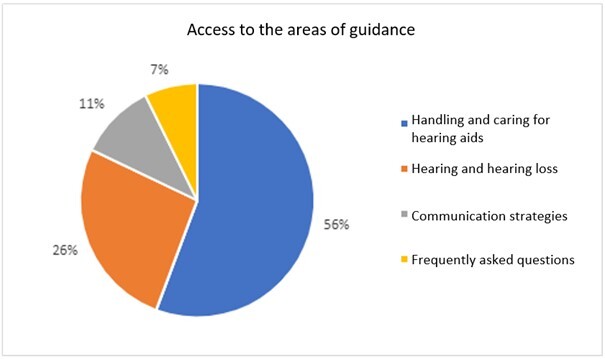
Distribution of Google Analytics results on the most accessed pages by individuals who participated in this study (N=43)

Google Analytics demonstrated that the most accessed pages belong to the areas of handling and caring for hearing aids and hearing and hearing loss. The results are shown below in Figure 1.

In the relationship between the number of accesses and interventions throughout the research, it was found that most interventions involved users in the experienced group, which mostly had older adults (Table 1).

**Table 1:** Distribution of analysis results of the relationship between the number of accesses and interventions by individuals who participated in this research, according to the new and experienced users’ groups (N=43)

Days	**General**		**New**		**Experienced**	
	**Correlation**	**p-value**	**Correlation**	**p-value**	**Correlation**	**p-value**
total	0.290	0.059	0.139	0.596	0.406	0.040*
30	0.233	0.133	0.362	0.153	0.256	0.206
90	0.279	0.169	0.177	0.497	0.167	0.415
180	0.168	0.280	-0.183	0.482	0.323	0.107
+180	0.331	0.030	0.394	0.117	0.315	0.118

* Spearman correlation

Most new and experienced participants reported being satisfied or very satisfied hearing aid users. They also liked remote healthcare and the system’s ease of use but said they would not indicate its use to everyone (Table 2, encompassing N = 40 because three research participants did not finish the questionnaire).

**Table 2:** Distribution of analysis results of system use by individuals who participated in the study (N = 40)

	Answer	N	%
Did you like receiving remote guidance as a complement to in-person appointments?	Yes	27	68%
	I did not need guidance; my fitting is fine.	4	10%
	No, I prefer it in person.	9	23%
Did you find the system easy to browse?	Yes	23	58%
	With someone else’s help.	12	30%
	No, I find the Internet too difficult.	5	13%
Would you recommend this form of healthcare to other patients?	Yes	24	60%
	Yes, but not to everyone.	16	40%
	No	0	0%

System usability was assessed to determine the subjective factors that impact system effectiveness and could be translated into actions to improve the users’ experience. SUS^(19)^ was applied to assess the usability of the system developed in the research, and its responses were analyzed according to amplification use, occupation, and age. Results are shown below in Table 3 (encompassing N = 41 because two participants did not finish the questionnaire).

**Table 3:** Distribution of SUS results analysis, answered by individuals who participated in the study, according to the groups of new and experienced users, younger and older adults, active and inactive (N = 41)

**Variable**	**Group**	**Mean**	**SD**	**Median**	**Minimum**	**Maximum**
						
Total	Participants	71.1	9.4	72.5	43	93
						
Age	Younger (< 60 years)	75.4	12.6	75	43	93
	Older (60 or more years)	70.7	7.6	72.5	53	85
						
Time of use	New	72.4	8.7	72.5	58	90
	Experienced	71.9	10	72.5	42	93
						
Professional activity	Inactive	72.08	8.3	72.5	53	85
	Active	72.07	10.3	72.5	43	93

**Figure 2:**
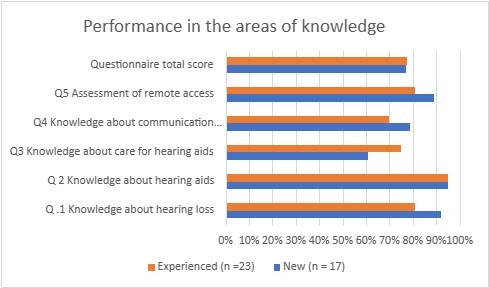
Distribution of answers to the questionnaires on the knowledge about system guidance by individuals who participated in the research, according to the groups of new and experienced users (n = 40)

The participants’ answers regarding knowledge of how to handle hearing aids are shown in Figure 2.

Caption: M1 = Initial moment of collection; M2 = Intermediate moment of collection; M3 = Final moment of collection

SSQ performance was analyzed per domain in different moments of the research, involving all participants. Results are shown below in Table 4.

**Table 4:** Distribution of results of the Speech Spatial and Qualities of Hearing Scale, according to the groups of new and experienced users in the different moments of the research (N=43)

**M**	**Domain**	**Group**	**Mean**	**Median**	**SD**	**Minimum**	**Maximum**	**p**
SSQ 1	Hearing speech	New	7.61	8.10	1.61	3.60	9.60	0.057
		Experienced	6.26	6.20	2.07	2.40	9.60	
	Spatial hearing	New	7.46	7.67	1.25	4.33	9.33	0.774
		Experienced	7.40	8.00	1.97	2.50	10.00	
	Qualities of hearing	New	7.56	7.50	1.34	5.25	9.75	0.190
		Experienced	6.83	6.96	1.77	3.25	9.50	
	Total	New	7.51	7.69	1.31	4.83	9.58	0.174
		Experienced	6.76	7.13	1.67	3.33	9.50	
SSQ 2	Hearing speech	New	7.63	7.60	1.40	5.00	9.40	**0.010**
		Experienced	5.86	6.40	2.00	2.20	8.20	
	Spatial hearing	New	7.31	7.67	1.61	4.33	9.67	0.415
		Experienced	6.73	7.00	2.26	2.67	10.00	
	Qualities of hearing	New	7.50	7.25	1.18	5.75	9.50	0.136
		Experienced	6.46	7.00	1.80	3.25	8.75	
	Total	New	7.51	7.50	1.29	5.67	9.42	0.07
		Experienced	6.21	6.67	1.85	2.75	8.73	
SSQ 3	Hearing speech	New	8.02	8.25	1.24	6.00	10.00	**0.049**
		Experienced	6.68	7.50	2.15	1.40	9.00	
	Spatial hearing	New	7.87	7.67	1.48	5.33	10.00	0.660
		Experienced	7.51	7.67	2.33	3.00	10.00	
	Qualities of hearing	New	7.95	8.00	1.36	6.00	10.00	0.300
		Experienced	7.20	7.75	1.68	3.67	9.50	
	Total	New	7.96	7.67	1.27	6.25	10.00	0.278
		Experienced	7.09	8.00	1.95	2.45	9.17	

* Student’s t-test

**Figure 3:**
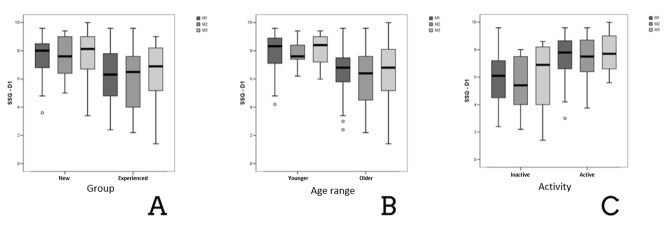
Boxplot with the values of the domain Hearing speech, according to the groups of users, divided by time of use (A), age range (B), and active or inactive professional activity (C), in the different moments of the research (N=43)

The analysis of the progress of SSQ performance regarding occupation and age did not find significant data. On the other hand, significant data were found in the analysis per time of experience (Figure 3).

The descriptive analysis regarding hearing aid use recorded and reported throughout the research is described in Table 5.

**Table 5:** Distribution of individuals who participated in the study according to the reported and recorded hours in the beginning and end of the research (N=43)

Group	Recorded use initial phase	Reported use initial phase	Reported use final phase
New	11.6	11.17	11.2
Experienced	9.2	10.61	11.39
Inactive	9.5	10.65	11.41
Active	10.78	11	11.26
Younger (< 60 years)	11.82	11.57	11.7
Older (60 or more)	9.38	10.48	11.07

## DISCUSSION

Good hearing aid performance requires that users be well instructed on how to handle and care for their devices, as well as communication strategies and speech-language-hearing follow-up. This study developed an online telemonitoring and teleguidance system for new and experienced hearing aid users. The impact of system use was analyzed by measuring the number of accesses, most accessed areas, system usability, and performance with devices throughout telemonitoring.

Group analysis showed significant results, with more access in the experienced group (Table 1). In an individual analysis of both new and experienced users, older adults accessed the system more often than adults. This may be due to difficulties retaining information as part of the aging process^(5)^, which requires them to review content to better understand the information because of episodic memory loss of specific facts^(25)^ Concerning time of use and occupation, no differences were found between the number of accesses, regardless of the time of use (experienced or new users) and occupation (active or inactive). These findings show the need for longitudinal follow-up on the users after the time of hearing aid fitting and reinforce the importance of making the reusable tool available, regardless of the time of experience.

The most accessed pages in the system include material with visual resources, which demonstrates the importance of this type of content in teleguiding hearing aid users (Figure 1). Routine topics (such as instructing on how to clean the hearing aids and change their filters and providing information on hearing and hearing loss) were the most consulted throughout the study by new and experienced users. This reinforces the importance of revisiting information provided in in-person consultations with reusable tools, allowing users and families to revise the information as often as needed, helping them better understand the information, and ensuring greater hearing aid use performance^(2,11)^.

Considering the total sample in relation to the analysis of system use by study participants (Table 2), most subjects said remote guidance was a positive, easy-to-browse complement to in-person consultations and that they would recommend this type of healthcare to other patients. Even though technology use by older adults faces resistance from both professionals and users, this research demonstrated significant tool use by this age group. The findings corroborate the literature that demonstrates that the online tool provides greater autonomy for users to manage hearing aid use, thus ensuring greater satisfaction^(2,11,12,15)^. The non-recommendation of this type of healthcare to everyone is also reported in the literature, which suggests its use by subjects familiarized with computers and who enjoy reading and obtaining information on their own^(12)^.

Despite the growing number of older adults who regularly use online tools, many older adults have little or no acquaintance with computer use. Using such instruments could be recommended to relatives and/or caregivers, besides the hearing aid users, to improve access to information, as patient-centered healthcare, along with the partnership with relatives, helps hearing aid users have fewer activity limitations and participation restrictions in their daily lives^(11,26)^. This study verified the importance of family participation in caring for and handling hearing aids.

SUS^(19)^ answers were analyzed to determine subjective factors that impact system effectiveness and could be translated into actions to improve user experience. SUS is a quick and easy-to-apply instrument, developed for superficial assessment to identify possible inconsistencies in the system^(19)^. The results were assessed according to amplification experience, occupation, and age (Table 3), and it was found that the mean performance in all analyses was above 70 points, which demonstrates good system usability. It was also found that subjects preferred accessing from a computer, which points to the positive decision of developing a system that can be accessed from different devices. The characterization of the sample, whose individuals were treated in a private clinic, may have made it easier to access the system and decide for using a computer. However, system usability still needs to be assessed considering those who access it from mobile phones.

Older adults tend to prefer using the computer instead of mobile phones because of its better readability, thanks to screen size. This makes it possible to democratically reach more hearing aid users, regardless of the device they use. The analysis of the system “I can hear, but I can't understand” based on SUS results proved it to be effective and efficient, ensuring user satisfaction and learning capacity. The possibility of revising the content asynchronously as often as needed collaborated to tolerance to errors, memorization, and flexibility, which are important usability components^(13,17,18)^.

It was possible to observe the analysis results of the user’s knowledge of hearing and handling hearing aids throughout the research (Figure 2). Experienced users initially had better results on how to care for the hearing aids than new users, whereas, at the end of the study, new and experienced users had similar results, close to 80% performance – whose improvement with remote guidance was not influenced by amplification experience, occupation, or age. These data agree with the study by Reese and Hnath-Chisolm^(27)^, in which new hearing aid users recognized on average 74% of the information on how to use and care for the devices after guidance. This is because online tools helped individuals implement behavioral changes in their activities of daily living, which positively increased the performance with hearing aid use, minimized the need for new interventions, and ensured greater satisfaction^(2,12)^.

SSQ performance analysis per domain in the three moments of the research shows positive responses in all domains, revealing progress in the use of new hearing aids. There was significant data in the domain of hearing speech, in which new users, who are also predominantly younger, performed better than the experienced group (Table 4). These results agree with those by Gatehouse and Noble^(28)^ and Moulin and Richard^(29)^, who verified that hearing speech is the domain with the worst scores when applied to groups with normal hearing, hearing loss, young people, or older adults.

The analysis of the domain of hearing speech divided by groups according to age, occupation, and time of use revealed significant improvements among young and active people in hearing speech (Figure 3). These data agree with the study by Moulin and Richard^(29)^, who observed that the effect of age and years at school influenced significantly the three questionnaire domains, with greater differences among young people and adults in the assessment of hearing speech than in the domains of spatial hearing and qualities of hearing.

The analysis of daily use (Table 5) showed an increase in the number of hours they used the device per day, especially among experienced users. These data agree with studies that indicate greater time of hearing aid use and increased satisfaction reported by users who consulted online material^(2,12)^. They were also observed by Nielsen and Carneiro^(30)^, who found increased management skills, time of use, and benefit from the hearing aids when new users received subsequent guidance.

Data in this study demonstrate the importance of a telemonitoring program for hearing aid users, regardless of the age or amplification experience^(2,12)^. Reusable material can provide valuable learning and improve knowledge to new users, ensuring better performance and the adequate use of hearing aids.

The study developed short guidance videos that were available in the system and were sent to participating individuals through a link to the YouTube channel “I can hear but I can't understand”. Once the videos were available, some users started changing the dome and filters in their hearing aids autonomously, which agrees with the study by Ferguson^(2)^, who demonstrated increased learning in practical skills to handle the devices after watching guidance videos.

Highly usable online tools as a complement to in-person consultation are an alternative to increase users’ access to professional guidance throughout their hearing rehabilitation, rather than only in the initial fitting process. Thus, patients remain close to their speech-language-hearing therapists to ensure quality guidance, avoid non-use, and improve performance with the hearing aids.

The coronavirus pandemic not only forced people to keep a distance from each other but also reinforced the importance of teleaudiology services, which proved to be appropriate for these therapists’ everyday clinical practice. The system developed in this study can provide an alternative model for services to hearing aid users, who can benefit from combined in-person and remote consultation, meeting the needs of patients who live far from the service or prefer remote hearing rehabilitation. Hence, it is increasingly important to create monitoring programs users can access easily, with patient-centered healthcare and tools that helped them self-manage their hearing loss.

The limitations of this study are related to the fact that the same person was both the clinician and researcher who collected research data, and patients may have been influenced to give favorable evaluations (social desirability bias). Another limitation is the lack of a comparator to find whether this system would have the same results in patients with different levels of educational attainment. It is important to improve the tool based on the results and conduct further studies related to the development of telemonitoring material for hearing aid users, aiming to improve their performance after hearing rehabilitation.

## CONCLUSION

The system “I can hear, but I can't understand” was developed as an effective tool to guide and telemonitor hearing aid users, increasing the time and regularity of device use, and improving the performance throughout the fitting process.

The system used by the pilot group proved to have great usability, ensuring use effectiveness, efficiency, and satisfaction, which demonstrates that online material that can be revisited is useful to ensure adherence to hearing aid use and improve hearing rehabilitation.
