# Supplementary material for: Development of an Internet-based system to guide and telemonitor hearing aid users
Source: Codas. 2023 Dec 4;35(6):e20220162. doi: 10.1590/2317-1782/20232022162en (PMC10750822; doi:10.1590/2317-1782/20232022162en)
Supplement: Translation

# Translation: Desenvolvimento de sistema baseado na internet para orientação e telemonitoramento de usuários de prótese auditiva

**DOI:** 10.1590/2317-1782/20232022162pt

**Authors:** Andrea Soares, Katia de Almeida

**Affiliations:** 1 Faculdade de Ciências Médicas da Santa Casa de São Paulo - FCMSCSP - São Paulo (SP), Brasil.

**Keywords:** Auxiliares de Audição, Idoso, Adulto, Aconselhamento à Distância, Intervenção Baseada em Internet, Telessaúde, Desenvolvimento Tecnológico, Satisfação do Paciente

## Abstract

**Objetivo:**

Desenvolver e verificar a usabilidade de um sistema baseado na internet para telemonitoramento e orientação do usuário de prótese auditiva bem como monitorar o desempenho de longo prazo em um grupo piloto.

**Método:**

O sistema Escuto, mas não entendo foi desenvolvido baseado em recomendações de literatura para layout, design e conteúdo de orientação e aconselhamento. Seguimos três etapas: planejamento, elaboração do design e conteúdo e teste piloto. A amostra foi formada por 43 adultos e idosos, com perda auditiva, de qualquer tipo e grau, uso regular de prótese auditiva de no mínimo 30 dias e no máximo 24 meses; com habilidade de leitura e sem evidências de comprometimentos cognitivos. Os indivíduos foram acompanhados por um período de oito a 12 meses. O desempenho dos usuários foi monitorado por meio do questionário Speech Spatial and Qualities of Hearing Scale. A usabilidade deste material foi avaliada com o questionário System Usability Scale.

**Resultados:**

Foi observada melhora de desempenho e aumento de uso diário autorrelatado das próteses auditivas após o período de orientação e telemonitoramento via sistema para todos os participantes da pesquisa. Em todas as análises da escala SUS foi possível observar o desempenho superior a 70 pontos, demonstrando a boa usabilidade do sistema. Na análise do desempenho do SSQ, nos três momentos da pesquisa, observou-se resposta positiva em todos os domínios, mostrando assim uma evolução com o uso das próteses auditivas, com dados significantes para o domínio Audição para a fala.

**Conclusão:**

A usabilidade do sistema foi considerada adequada pelos indivíduos participantes do estudo.

## INTRODUÇÃO

A adaptação da prótese auditiva é um processo múltiplo e complexo, que não se limita apenas ao período de testes e a escolha do dispositivo. Muitas pesquisas demonstram que ausência de orientação e acompanhamento inadequado implicará no abandono do uso das próteses auditivas^(1)^. Estima-se que a não utilização de próteses auditivas - já adquiridas - em adultos alcance até 24% dos usuários^(2,3)^.

Diversos usuários novos ou experientes relatam dificuldades em usar os dispositivos, insatisfação no uso e esforço para reter as orientações recebidas e alguns sujeitos não procuram auxílio, pois não percebem que estão enfrentando problemas ou que podem obter melhores resultados com o uso da amplificação^(4)^. As queixas mais comuns entre os usuários de próteses auditivas estão relacionadas ao uso do telefone, compreensão de fala no ruído, colocação correta da pilha, limpeza, identificação do lado do dispositivo, cuidados em relação a umidade e como ligar^(4,5)^. Esses problemas de manuseio são frequentemente considerados como os de maior impacto no sucesso da adaptação e os mais fáceis de solucionar^(6)^.

Para melhorar a experiência e aumentar a efetividade no processo de adaptação das próteses auditivas, é essencial que os fonoaudiólogos forneçam treinamento adequado, aconselhamento e monitoramento periódico para todos os indivíduos. Cabe ressaltar, que além de explicações verbais, os profissionais devem apresentar material escrito com linguagem apropriada levando em consideração a capacidade de leitura do indivíduo^(7)^, além dos princípios de usabilidade em *software educativo* ^(8)^.

Acompanhar o desempenho do usuário é essencial para garantir a boa adaptação das próteses auditivas. Para avaliar a autopercepção frente às dificuldades auditivas, limitações de funcionalidade e restrições de participação, bem como benefício e satisfação do usuário, são aplicados os questionários de autoavaliação. O uso dessas ferramentas, no atendimento, é um diferencial que pode trazer muitas informações qualitativas que auxiliam no processo de adaptação das próteses auditivas. Entre os questionários mais utilizados atualmente encontramos o *Speech, Spatial and Qualities of Hearing Scale* - SSQ. O SSQ é uma medida de autorrelato de “incapacidade auditiva”, que tem como objetivo avaliar a participação, do paciente, em atividades auditivas, experiências subjetivas e quantificar as inabilidades de escuta em situações de comunicação, como a audição direcional, facilidade de escuta e clareza de sons.

Os modelos tradicionais de prestação de serviços de reabilitação auditiva se concentram em testes presenciais, com ajustes dos dispositivos e aconselhamento que requerem várias visitas ao local de atendimento. Assim, pacientes que possuem dificuldades de comparecer presencialmente nas consultas, deixam de receber o acompanhamento adequado. Uma alternativa para aumentar a acessibilidade da orientação é o uso da telessaúde. Esse atendimento pode ser oferecido ao paciente que está perto do profissional de saúde, mas escolhe o atendimento não presencial como uma opção de prestação de serviço por conveniência. O atendimento híbrido ao usuário, acrescentando o teleatendimento ao presencial, também tem demonstrado eficácia e satisfação dos pacientes, tanto para validação das ferramentas, como para reabilitação auditiva com foco na satisfação e experiências dos pacientes^(9)^ com resultados semelhantes para usuários novos e experientes, independente da modalidade do atendimento^(10)^.

A telessaúde reduz custos de orientação, permite melhor acesso aos cuidados e melhor resultados de satisfação ao paciente usuário de prótese auditiva. Aumenta a absorção e a eficácia da orientação da prática clínica, especialmente em termos de desenvolvimento tecnológico, validação técnica e clínica, otimiza estratégias para prestação de serviços^(11)^ e fornece um teleatendimento centrado no paciente^(2,12-15)^.

Para avaliar a qualidade da experiência do usuário ao interagir com a tecnologia temos a usabilidade^(16)^, que verifica se um produto pode ser usado pelos usuários para atingir objetivos especificados com efetividade, eficiência e satisfação^(17)^. Nielsen^(18)^ definiu cinco componentes de qualidade para a usabilidade: aprendizagem, eficiência, capacidade de memorização, tolerância a erros e satisfação. Para Brooke^(19)^ os parâmetros de usabilidade como efetividade, eficiência e capacidade de aprendizagem devem ser medidos ou quantificados de forma pontual. Já as medidas subjetivas (satisfação do usuário) são mais genericamente avaliadas, por questionários ou escalas de atitude gerais, como o SUS - *System Usability Scale* (Escala de Usabilidade de Sistema).

Os testes de usabilidade fornecem *insights* valiosos sobre as experiências e o processo de uso do material desenvolvido. Também podem ser usados para explicar os resultados de medidas de resultado como benefício e satisfação autorrelatados^(13,19)^. Muitas discussões sobre inovações em saúde auditiva se concentraram nas necessidades não atendidas do paciente e em recursos para atendê-las, mas nenhuma discussão sobre tecnologia auditiva estará completa até que consideremos as competências da pessoa que usará a tecnologia^(13)^.

Frente ao exposto, esse estudo teve como objetivo desenvolver e verificar a usabilidade de um sistema baseado na internet para telemonitoramento e orientação do usuário de prótese auditiva bem como monitorar o desempenho de longo prazo em um grupo piloto.

## MÉTODO

Neste estudo seguimos três etapas: planejamento, elaboração do design e conteúdo e teste piloto. A escolha por desenvolver um sistema *online* e não um aplicativo foi baseada não apenas no alto custo do aplicativo, mas também na dificuldade de manter a atualização dos materiais de orientação e em relação ao sistema operacional dos telefones. O sistema pode ser acessado em computadores ou *smartphones* possibilitando assim o acesso a um maior número de usuários.

O material desenvolvido tem como objetivo ser uma ferramenta reutilizável, com informações curtas, visuais e interativas. Todo o material foi elaborado baseado em princípios de usabilidade e design, contemplando temas relacionados a orientação, o aconselhamento e o seguimento de adultos e idosos deficientes auditivos usuários de próteses auditivas.

O sistema recebeu o nome Escuto, mas não entendo e fica acessível por meio do site “Escuto mas não entendo” e está em registro no INPI (Instituto Nacional da Propriedade Industrial) com número de processo 512022001680-0. Na página inicial foram elaborados 6 textos de orientação e na área restrita foram elaborados 26 textos distribuídos nas seguintes divisões: 1. Espaço institucional; 2. Espaço para inserção de textos informativos, denominado Notícias; 3. Área restrita, na qual o paciente é cadastrado pelo responsável pelo atendimento; 4. Espaço do Paciente, com possibilidade de troca de mensagens, de forma assíncrona, entre profissional e paciente; 5. Área de autoavaliação: na qual o paciente responde o Questionário SSQ-12; e 6. Área de orientação. O conteúdo de orientação do sistema só pode ser acessado por meio de *login* e senha do paciente cadastrados, respeitando as diretrizes da Lei Geral de Proteção de Dados (LGPD).

A área de Orientação foi dividida em quatro seções: Audição e perda de audição; Manipulação e cuidados com os aparelhos auditivos; Estratégias de comunicação e Dúvidas Frequentes. Foram elaboradas 29 imagens (desenhos, ilustrações e fotografias) e 8 vídeos curtos, com menos de um minuto cada, para ilustrar os 26 textos de orientação, com intuito de facilitar a compreensão do usuário buscando aumentar a leiturabilidade do material de orientação elaborado. Para que os vídeos pudessem ter acesso público foi criado o canal “Escuto mas não entendo” na plataforma *YouTube*. A terminologia “aparelhos auditivos” foi utilizada em todos os materiais de orientação direcionados aos usuários de próteses auditivas por ser mais popular e de conhecimento do público leigo.

O projeto foi analisado e aprovado pelo Comitê de Ética em Pesquisa da Santa casa de São Paulo, sob o número 3.443.374. Os indivíduos que aceitaram participar voluntariamente do estudo assinaram o termo de consentimento livre e esclarecido - TCLE. A coleta de dados foi realizada em uma clínica particular na cidade de São Paulo. Participaram 43 indivíduos, com idades entre 27 e 87 anos, de ambos os sexos, perda auditiva bilateral ou unilateral, de qualquer tipo e grau, classificada de acordo com a referência da OMS^(20)^. Os critérios de inclusão adotados foram: usuários de prótese auditiva há no máximo 24 meses, alfabetizados; novos usuários, com no mínimo 30 dias de uso; ausência de problemas visuais importantes; boa habilidade de leitura, sem evidências de comprometimentos cognitivos. Os critérios de exclusão adotados foram: impossibilidade de acesso à internet e não fazer uso regular da prótese auditiva.

Durante a consulta presencial, foi realizada uma anamnese buscando obter informações sobre escolaridade, atividade profissional, uso regular das próteses auditivas e dificuldades associadas.

Para o estudo foi considerado como ativo o indivíduo que exerce alguma profissão remunerada e inativo os demais sujeitos. Ainda nesta consulta foi avaliado o tempo de uso diário registrado no *software* e declarado pelo paciente. Todos os pacientes passaram pelo mesmo protocolo de adaptação a fim de garantir a adequada programação das próteses auditivas no início da coleta.

O desempenho dos usuários foi monitorado por meio do questionário SSQ na versão reduzida com 12 questões traduzida para o português brasileiro^(21,22)^. Usuários experientes responderam ao questionário com base no desempenho com as próteses auditivas no momento, o que chamamos de SSQ-1. Os novos usuários responderam o SSQ-1, com base no desempenho com o uso das próteses auditivas, após 30 dias da adaptação concluída. Aqueles que receberam intervenção responderam após a teleconsulta o questionário SSQ, que chamamos de SSQ-2. Ao final deste período de telemonitoramento foi solicitado que o indivíduo respondesse novamente o SSQ, por meio do sistema, para avaliar o benefício e satisfação após este período, a esta aplicação do questionário chamamos de SSQ-3. Foi utilizada uma escala com pontuação de 0 a 10, sendo que 10 indicava perfeito desempenho e 0 indicava grande dificuldade no momento da resposta.

Todos os participantes foram acompanhados por um período de no mínimo oito e no máximo 12 meses a fim de avaliar a efetividade do material desenvolvido, usando uma abordagem participativa durante todo o período de telemonitoramento. Todos os participantes do estudo foram orientados a usar a sessão de mensagens assíncronas sempre que necessário relatar dificuldades ou esclarecer dúvidas. O profissional respondeu as mensagens de forma assíncrona enviando materiais de orientação do sistema, de forma direcionada, e quando necessário agendou atendimento por meio de teleconsultas com vídeos chamadas.

No final deste período foi registrado o tempo de uso diário das próteses auditivas, declarado pelo paciente, que foi atendido presencialmente quando necessário. O tempo de uso final, medido pelo registro de dados (*data logging)*, não pôde ser medido devido ao período de isolamento e restrições aplicados neste período da pandemia.

Para analisar a usabilidade do sistema desenvolvido na pesquisa foi enviado para cada participante o questionário com a Escala de Satisfação de Usabilidade (SUS)^(19)^, e um questionário para relato de qual a área do sistema que mais foi acessada pelos usuários. Ambos os instrumentos foram enviados ao término do período de telemonitoramento, por meio da plataforma *Google Forms*. O objetivo desta análise foi avaliar a exploração do sistema (realização das tarefas e facilidade no acesso), efetividade de uso (capacidade para encontrar informações dentro do sistema), verificar a compreensão das orientações de uso descritas no sistema, eficiência de uso (questões sobre os níveis de dificuldade e conforto ao usar as informações do sistema) e por último a satisfação de uso (questões relacionadas a reações subjetivas dos usuários após usar o sistema).

A análise da escala SUS foi realizada de acordo com o proposto por Lewis e Sauro^(23)^ analisando o questionário completo e não por questões separadamente. A pontuação de cada item, pode variar de 0 a 4. Para itens com palavras positivas (números ímpares), a contribuição da pontuação é a posição da escala menos 1. Para itens com palavras negativas (pares números), a contribuição da pontuação é 5 menos a posição da escala. Para obter a pontuação geral, multiplica-se a soma das contribuições da pontuação do item por 2,5 produzindo um índice pode variar de 0 (usabilidade percebida muito ruim) a 100 (usabilidade percebida excelente), sendo que uma pontuação de 68 é considerada média e de 80 pontos usabilidade acima da média^(23,24)^.

Para verificar as páginas do sistema mais acessadas durante o período do telemonitoramento, e dispositivos utilizados no acesso, foram extraídos os dados do *Google Analytics.* Para saber se as respostas eram semelhantes a percepção dos usuários foi enviado um questionário, desenvolvido para esta pesquisa, com quatro itens correspondentes as áreas do sistema (Audição e perda de audição; Manipulação e cuidados com os aparelhos auditivos; Estratégias de comunicação e Dúvidas Frequentes). O usuário deveria indicar qual a área mais acessada durante o período de telemonitoramento, sendo possível selecionar apenas uma das áreas descritas. Para essa análise os participantes foram alocados em grupos, de acordo com idade (jovens = até 60 anos e idosos = 60 anos ou mais), atividade profissional (ativos = 23 usuários e inativos = 20 usuários) e tempo de uso das próteses auditivas (novos usuários = 17 e usuários experientes = 26). Foi realizada uma análise descritiva para variáveis qualitativas: sexo, escolaridade, grau e tipo da perda auditiva, atividade profissional, características da prótese auditiva. Os testes estatísticos foram aplicados por meio do programa SPSS 25.0.

## RESULTADOS

A amostra foi composta por 43 indivíduos distribuídos em dois grupos, de acordo com a experiência com as próteses auditivas: 26 usuários experientes (14 mulheres com média de idade de 53,8 anos e 12 homens com média de idade de 46,2 anos), com até 2 anos de uso e um grupo de 17 novos usuários (8 mulheres com média de idade de 47,1 anos e 9 homens com média de idade de 52,9 anos). Não foram observadas diferenças estatisticamente significantes, quando comparados os grupos segundo as variáveis sexo e idade (p=0,663 Qui-quadrado). A escolaridade dos sujeitos variou de ensino médio completo a pós-graduação, sem diferenças significantes entre os grupos (p=0,461).

Os limiares auditivos da amostra foram similares entre os grupos, em relação a lateralidade da perda auditiva (p=1, teste Exato de Fisher) e de uso da prótese auditiva (p=0,376, teste Exato de Fisher) (unilateral x bilateral) e tipo de perda auditiva na orelha direita (p=0,227, teste Qui-quadrado) e na orelha esquerda (p=0,348, teste Qui-quadrado) configurando uma amostra com perda auditiva predominante neurossensorial e em ambas as orelhas e com adaptação de prótese auditiva bilateral.

No que se refere a atividade profissional houve diferenças estatisticamente significantes entre os grupos p= 0,002, na análise com teste Qui-quadrado entre os ativos e inativos. O grupo de usuários experientes foi composto em sua maioria por indivíduos inativos profissionalmente 65,4% (n=17) e o grupo de novos usuários de indivíduos ativos profissionalmente 82,4% (n=14).

O acesso ao sistema foi realizado em 91% das vezes pelo próprio usuário independentemente da idade e 9% por familiares ou cuidadores. A maior parte do acesso foi realizada pelo computador (69,95%) e celulares (29,58%), apenas uma pequena parte do acesso foi por tablet (0,46%) durante todo o período de telemonitoramento.

Na análise do *Google Analytics* foi observado que as páginas mais acessadas fazem parte da área “Manipulação e Cuidados com os Aparelhos Auditivos” e da área “Audição e Perda de Audição”. Os resultados são apresentados a seguir na Figura 1.

**Figura 1:** Distribuição dos resultados obtidos na análise do Google Analytics sobre as páginas mais acessadas pelos indivíduos que participaram do estudo (N=43)

Na relação entre o número de acessos e intervenções ao longo da pesquisa, observou-se que o maior número de intervenções foi dos usuários do grupo experiente, que é composto em sua maioria de indivíduos idosos (Tabela 1).

**Tabela 1:** Distribuição dos resultados obtidos na análise da relação aos números de acesso e de intervenção realizados pelos indivíduos que participaram da pesquisa, de acordo com os grupos de usuários novos ou experientes (N=43)

Dias	**Geral**		**Novo**		**Experiente**	
	**Correlação**	**Valor p**	**Correlação**	**Valor p**	**Correlação**	**Valor p**
total	0,290	0,059	0,139	0,596	0,406	0,040*
30	0,233	0,133	0,362	0,153	0,256	0,206
90	0,279	0,169	0,177	0,497	0,167	0,415
180	0,168	0,280	-0,183	0,482	0,323	0,107
+180	0,331	0,030	0,394	0,117	0,315	0,118

* Correlação de *Spearman*

Os usuários novos e experientes declararam em sua maioria se sentir satisfeito ou muito satisfeito como usuários de próteses auditivas. Relataram ainda gostar do atendimento remoto e da facilidade de uso do sistema, mas consideraram que não indicariam o uso a qualquer pessoa (Tabela 2), contemplando N=40, porque três participantes da pesquisa não completaram o questionário.

**Tabela 2:** Distribuição dos resultados obtidos na análise do uso do sistema pelos indivíduos que participaram do estudo (N=40)

	Resposta	N	%
Você gostou de ser orientado de forma remota como complemento das consultas presenciais?	Sim	27	68%
	Não precisei de orientação, estou bem adaptado	4	10%
	Não, prefiro presencial	9	23%
Você achou fácil navegar no sistema?	Sim	23	58%
	Com a ajuda de outra pessoa	12	30%
	Não, acho a internet muito difícil	5	13%
Você recomendaria essa forma de atendimento para outros pacientes?	Sim	24	60%
	Sim, mas não para qualquer pessoa	16	40%
	Não	0	0%

A usabilidade do sistema foi avaliada com o intuito de determinar os fatores subjetivos que impactassem a efetividade do sistema e que pudessem ser traduzidos em ações que aperfeiçoassem a experiência do uso. Foi aplicada para avaliar a usabilidade do sistema desenvolvido na pesquisa a escala de usabilidade - *System Usability Scale* - SUS^(19)^ e as respostas foram analisadas de acordo com experiência com o uso da amplificação, atividade laboral e idade. Os resultados são apresentados a seguir na Tabela 3, contemplando N=41, porque dois participantes da pesquisa não completaram o questionário SUS.

**Tabela 3:** Distribuição dos resultados obtidos na análise do questionário SUS respondido pelos indivíduos que participaram do estudo, de acordo com os grupos de usuários novos ou experientes, jovens ou idosos, ativos ou inativos (N=41)

**Variável**	**Grupo**	**Média**	**DP**	**Mediana**	**Mínimo**	**Máximo**
						
Total	participantes	71,1	9,4	72,5	43	93
						
idade	Jovem (< 60 anos)	75,4	12,6	75	43	93
	Idoso (60 anos ou mais)	70,7	7,6	72,5	53	85
						
tempo de uso	Novo	72,4	8,7	72,5	58	90
	Experiente	71,9	10	72,5	42	93
						
atividade profissional	Inativo	72,08	8,3	72,5	53	85
	Ativo	72,07	10,3	72,5	43	93

As respostas dos indivíduos participantes da pesquisa em relação ao conhecimento sobre uso de manipulação das próteses auditivas, estão demonstradas na Figura 2:

**Figura 2:** Distribuição das respostas aos questionários sobre o conhecimento em relação as áreas de orientação do sistema pelos indivíduos que participaram da pesquisa, de acordo com os grupos de usuários novos ou experientes (N=40)

Legenda: Legenda: M1 = Momento incial da coleta; M2 = Momento intermediário da coleta; M3 = Momento final da coleta

Foi analisado o desempenho do SSQ, por domínio, em diferentes momentos da pesquisa, para todos os indivíduos participantes. Os resultados são apresentados a seguir na Tabela 4:

**Tabela 4:** Distribuição dos resultados obtidos na aplicação do questionário *Speech Spatial and Qualities of Hearing Scale*, de acordo com os grupos de usuários novos ou experientes, de acordo com o momento da pesquisa (N=43)

**M**	**Domínio**	**Grupo**	**Média**	**Mediana**	**DP**	**Mínimo**	**Máximo**	**p**
SSQ 1	Audição para a fala	Novo	7,61	8,10	1,61	3,60	9,60	0,057
		Experiente	6,26	6,20	2,07	2,40	9,60	
	Audição espacial	Novo	7,46	7,67	1,25	4,33	9,33	0,774
		Experiente	7,40	8,00	1,97	2,50	10,00	
	Qualidades da audição	Novo	7,56	7,50	1,34	5,25	9,75	0,190
		Experiente	6,83	6,96	1,77	3,25	9,50	
	Total	Novo	7,51	7,69	1,31	4,83	9,58	0,174
		Experiente	6,76	7,13	1,67	3,33	9,50	
SSQ 2	Audição para a fala	Novo	7,63	7,60	1,40	5,00	9,40	**0,010**
		Experiente	5,86	6,40	2,00	2,20	8,20	
	Audição espacial	Novo	7,31	7,67	1,61	4,33	9,67	0,415
		Experiente	6,73	7,00	2,26	2,67	10,00	
	Qualidades da audição	Novo	7,50	7,25	1,18	5,75	9,50	0,136
		Experiente	6,46	7,00	1,80	3,25	8,75	
	Total	Novo	7,51	7,50	1,29	5,67	9,42	0,07
		Experiente	6,21	6,67	1,85	2,75	8,73	
SSQ 3	Audição para a fala	Novo	8,02	8,25	1,24	6,00	10,00	**0,049**
		Experiente	6,68	7,50	2,15	1,40	9,00	
	Audição espacial	Novo	7,87	7,67	1,48	5,33	10,00	0,660
		Experiente	7,51	7,67	2,33	3,00	10,00	
	Qualidades da audição	Novo	7,95	8,00	1,36	6,00	10,00	0,300
		Experiente	7,20	7,75	1,68	3,67	9,50	
	Total	Novo	7,96	7,67	1,27	6,25	10,00	0,278
		Experiente	7,09	8,00	1,95	2,45	9,17	

* Teste t-Student

A análise da relação da evolução do desempenho no SSQ em relação à atividade profissional e idade não apresentou dados significantes. Foram observados dados significantes na análise por tempo de experiência (Figura 3).

**Figura 3:** BoxPlot dos valores do domínio Audição para a fala, de acordo com os grupos de usuários, divididos por tempo de uso (A), faixa etária (B) e atividade profissional ativa ou inativa (C), nos diferentes momentos da pesquisa (N=43)

A análise descritiva em relação ao uso registrado e declarado das próteses auditivas ao longo da pesquisa é descrita na Tabela 5.

**Tabela 5:** Distribuição dos indivíduos que participaram do estudo, de acordo com horas de uso declaradas e registradas no início e no final da pesquisa (N=43)

Grupo	Uso Registrado fase inicial	Uso Declarado fase inicial	Uso Declarado fase final
Novo	11,6	11,17	11,2
Experiente	9,2	10,61	11,39
Inativo	9,5	10,65	11,41
Ativo	10,78	11	11,26
Jovem (< 60 anos)	11,82	11,57	11,7
Idoso (60 ou mais)	9,38	10,48	11,07

## DISCUSSÃO

Para um bom desempenho com as próteses auditivas é importante que o usuário seja bem orientado em relação a manipulação e cuidados com as próteses auditivas, estratégias de comunicação e acompanhamento fonoaudiológico. Neste estudo desenvolveu-se um sistema *online* para telemonitoramento e teleorientação de usuários, novos e experientes, de prótese auditiva. Para mensurar o impacto do uso do sistema foram analisados o número de acessos, áreas mais acessadas, usabilidade do sistema e desempenhos com dispositivo durante o período de telemonitoramento.

Na análise por grupos, observamos resultados significantes com maior acesso no grupo de experientes (Tabela 1). Em análises individuais, tanto no grupo de usuários novos como de experientes, os idosos acessaram mais vezes que os adultos. Esse fato pode estar relacionado a dificuldade de retenção de informações, decorrente do processo de envelhecimento^(5)^ que torna necessária a revisão de conteúdo para melhor compreensão das informações devido à perda de memória episódica, para fatos específicos^(25)^. Em relação as variáveis tempo de uso e atividade profissional, não foram observadas diferenças entre o número de acessos, independente do tempo de uso (experientes e novos usuários) e atividade profissional (ativos e inativos). Esses achados demonstram a necessidade de acompanhamento longitudinal dos usuários após o período da adaptação das próteses auditivas e reforçam a importância da disponibilização de ferramenta reutilizável, independente do tempo de experiência.

As páginas mais acessadas no sistema incluem materiais com recursos visuais, o que demonstra a importância desse tipo de material na teleorientação aos usuários de próteses auditivas (Figura 1). Temas rotineiros como aqueles que orientam como limpar as próteses auditivas, trocar o filtro e informações sobre audição e perda de audição foram os mais consultados ao longo do estudo tanto por usuários novos como experientes. O que reforça a importância de revisitar as informações fornecidas em consultas presenciais, por meio de ferramentas reutilizáveis, permitindo ao usuário e familiares a revisão da informação, quantas vezes julgarem necessário e levam a uma melhor compreensão das informações prestadas e o maior desempenho no uso da prótese auditiva^(2,11)^.

Considerando a amostra total em relação a análise do uso do sistema pelos participantes do estudo (Tabela 2), a maioria dos sujeitos relataram como positiva a orientação remota como complemento das consultas presenciais, e fácil navegação ao sistema e recomendariam essa forma de atendimento para outros pacientes. Boa parte da amostra referiu recomendar a forma de atendimento, mas não para qualquer pessoa. Embora o uso de tecnologia por indivíduos idosos ainda receba uma resistência tanto de profissionais como de usuários, a pesquisa demonstrou significativa participação deste grupo no uso da ferramenta. Os achados corroboram a literatura compulsada que demonstra que a ferramenta *online* proporciona maior autonomia para os usuários gerenciarem o uso das suas próteses auditivas o que gera assim, maior satisfação^(2,11,12,15)^. A não recomendação desse tipo de atendimento para todos os usuários também é reportada na literatura que sugere a utilização por sujeitos que tenham familiaridade com uso de computadores, gostam de ler e de obter informações sozinhos^(12)^.

Apesar do crescente número de idosos que utilizam regularmente as ferramentas *online*, ainda há uma boa parte da população idosa que tem pouca ou nenhuma familiaridade com o uso de computadores. Para melhorar o acesso às informações, o uso desses instrumentos poderia ser recomendado aos familiares e/ou cuidadores, além dos próprios usuários de próteses auditivas, pois o atendimento centrado no indivíduo em conjunto com a parceria com os familiares faz com que os usuários de prótese auditiva apresentem menor limitação em atividades e restrição de participação em vida diária^(11,26)^. Neste estudo foi possível observar a importância da participação dos familiares no cuidado e manipulação das próteses auditivas.

Com o intuito de determinar os fatores subjetivos que impactassem a efetividade do sistema e que pudessem ser traduzidos em ações que aperfeiçoassem a experiência do uso foram analisadas as respostas por meio da escala de usabilidade - *System Usability Scale* - SUS^(19)^. O SUS é um instrumento criado para avaliação superficial, que visa identificar possíveis inconsistências no sistema de forma rápida e fácil aplicação^(19)^. Os resultados foram avaliados de acordo com experiência com o uso da amplificação, atividade laboral e idade (tabela 3) e foi possível observar que para todas as análises a média de desempenho foi superior a 70 pontos, demonstrando a boa usabilidade do sistema. Também foi observado o acesso preferencialmente pelo computador, o que indica a assertividade em desenvolver um sistema que possa ser acessado de diferentes dispositivos. A caracterização da amostra, com indivíduos atendidos em clínica particular, talvez tenha facilidade o acesso e escolha pelo acesso via computador. Contudo, ainda é preciso avaliar a usabilidade desse sistema para aqueles que acessarem via celular.

Os idosos tendem a preferir o acesso pelo computador em vez de pelo celular por conta da melhor leiturabilidade proporcionada pelo tamanho da tela, o que permite atingir um maior número de usuários de próteses auditivas, de forma mais democrática, independente do dispositivo de acesso. A análise do sistema *Escuto, mas não entendo* pelos resultados obtidos na escala SUS, demonstrou boa efetividade, eficiência, satisfação dos usuários e capacidade de aprendizagem. A possibilidade de rever o conteúdo tantas vezes quantas forem necessárias, de forma assíncrona, colaborou com a tolerância ao erro, memorização e flexibilidade que são importantes componentes de usabilidade^(13,17,18)^.

Foi possível observar o resultado da análise do conhecimento sobre audição e manipulação das próteses auditivas dos usuários ao longo do tempo da pesquisa (Figura 2). Inicialmente, os usuários experientes apresentaram melhor resultado sobre os cuidados com as próteses que os novos usuários. Ao final do estudo, o desempenho de novos usuários e experientes foi similar, próximo de 80% de desempenho, não sendo influenciado pela experiência com o uso da amplificação, atividade laboral ou idade na melhora do desempenho com o uso de orientação remota. Estes dados concordam com o estudo de Reese e Hnath-Chisolm^(27)^ no qual novos usuários de próteses auditivas conseguiram reconhecer, em média, 74% das informações sobre o uso e cuidados com o dispositivo, após orientação. Isto deve-se ao fato de que, com o uso de ferramentas *online*, os indivíduos conseguiram implementar mudanças comportamentais em suas atividades diárias, o que aumentou positivamente o desempenho com o uso das próteses auditivas, minimizou a necessidade de novas intervenções e garantiram aumento da satisfação^(2,12)^.

Na análise de desempenho do SSQ, dividida por domínios, nos três momentos da pesquisa, observou-se uma resposta positiva em todos os domínios, mostrando assim uma evolução com o uso das novas próteses auditivas, com dados significantes para o domínio Audição para a fala, com melhor desempenho para os usuários novos, que também são predominantemente mais jovens que o grupo experiente (tabela 4). Tais resultados são coincidentes com os de Gatehouse e Noble^(28)^, Moulin e Richard^(29)^ que constataram que Audição para Fala é o domínio com pior pontuação quando aplicado em grupos com audição normal, com perda auditiva, em jovens ou idosos.

A análise do domínio Audição para a Fala, dividida por grupos de acordo com a idade, atividade profissional e tempo de uso, apresentou melhora significante para os jovens e os ativos no domínio Audição para a Fala (Figura 3). Estes dados concordam com o estudo de Moulin e Richard^(29)^, em que observaram que o efeito da idade e anos de escolaridade, tiveram influência significante nos três domínios do questionário, com maiores diferenças entre jovens e adultos na avaliação do domínio Audição para Fala do que nos domínios Audição Espacial e Qualidades da Audição.

A análise do uso diário (Tabela 5), observamos um aumento de horas de uso diário, principalmente para usuários experientes. Esses dados concordam com os estudos que relacionam maior tempo de uso das próteses auditivas e aumento na satisfação relatados por usuários que utilizam material *online* como consulta^(2,12)^; bem como observados por Nielsen e Carneiro^(30)^ que encontraram aumento das habilidades de manuseio, tempo de uso e benefício obtido com as próteses auditivas quando novos usuários são expostos a orientações subsequentes.

Os dados do presente estudo demonstram a importância de um programa de telemonitoramento aos usuários de próteses auditivas, independentemente da idade do indivíduo ou experiência com o uso da amplificação^(2,12)^. Um material reutilizável pode fornecer um aprendizado valioso e aprimoramento do conhecimento a novos usuários, proporcionando melhor desempenho no uso adequado das próteses auditivas.

No estudo foram elaborados vídeos curtos de orientação que ficaram disponíveis dentro do sistema e foram enviados aos indivíduos participantes por meio do *link* do canal *Escuto mas não entendo* do YouTube. Com os vídeos acessíveis, parte dos usuários passaram a trocar a oliva e filtros das suas próteses auditivas de forma autônoma, concordando com o estudo de Ferguson^(2)^ que demonstrou aumento no aprendizado e habilidades práticas relacionadas a manipulação dos dispositivos após uso de vídeos de orientação.

O uso de ferramentas *online*, de fácil usabilidade, como complemento da consulta presencial, são uma alternativa para aumentar a acessibilidade dos usuários a orientação profissional durante todo o período da reabilitação auditiva, e não só durante o processo inicial de adaptação, mantendo a proximidade do paciente com o fonoaudiólogo de forma a garantir qualidade de orientação, evitar o abandono e melhorar o desempenho com as próteses auditivas.

A pandemia pelo coronavírus, não apenas forçou as pessoas a ficarem distantes umas das outras, mas também reforçou a importância dos serviços de teleaudiologia que se mostraram ainda mais pertinentes de fazer parte da prática clínica e do dia a dia do fonoaudiólogo. O sistema desenvolvido pode oferecer um modelo alternativo de prestação de serviços ao usuário de próteses auditivas, que pode se beneficiar com a combinação da consulta presencial e não presencial, atendendo as necessidades dos pacientes que moram longe do serviço ou por preferirem um atendimento de reabilitação auditiva de maneira não presencial. Desta forma, torna-se cada vez mais importante criar programas de monitoramento de fácil acesso aos usuários, por meio de atendimento centrado no paciente com ferramentas que permitam o autogerenciamento da perda auditiva pelo usuário.

As limitações do presente estudo estão relacionadas ao fato de que a mesma pessoa atuou como clínico e pesquisador que coletou os dados da pesquisa, e os pacientes podem ter sido influenciados a fornecer avaliações favoráveis ​​(viés de desejabilidade social). Outra limitação é a falta de um comparador para saber se este sistema apresentaria os mesmos resultados em pacientes com diferentes níveis educacionais. Incentiva-se o aprimoramento da ferramenta com base nos resultados obtidos e recomenda-se mais estudos relacionados ao desenvolvimento de material para telemonitoramento ao usuário de prótese visando a melhora no desempenho destes indivíduos após a reabilitação auditiva.

## CONCLUSÃO

O sistema *Escuto, mas não entendo* foi desenvolvido como uma ferramenta efetiva para orientação e telemonitoramento do usuário de próteses auditivas, aumentando tempo e constância de uso dos dispositivos, com melhora de desempenho ao longo do processo de adaptação.

O sistema, utilizado pelo grupo piloto, mostrou-se de fácil usabilidade, garantindo a efetividade, eficiência e satisfação no uso, o que demonstra que materiais *online* que podem ser revisitados são úteis para garantir a aderência ao uso da prótese auditiva e melhora da reabilitação auditiva.
